# Online environments and women's health: an industry-academic public health research partnership to improve health inequities

**DOI:** 10.3389/fpubh.2023.1176198

**Published:** 2023-07-14

**Authors:** Vanessa Boland Edouard, Malu Foley, Allegra Gordon, Susan Garfield, Leyla Dincer, Kimberly M. Nelson

**Affiliations:** ^1^Idea Hub, Boston University School of Public Health, Boston, MA, United States; ^2^Ernst & Young LLP, Boston, MA, United States; ^3^Department of Community Health Sciences, Boston University School of Public Health, Boston, MA, United States

**Keywords:** academic-industry partnership, innovation, impact, population health, women's health

## Introduction

Academic-industry partnerships in public health are rare and present an opportunity to deepen our understanding of health inequities and improve the health of populations. The COVID-19 pandemic showed that industry and public health are inextricably interlocked: industry decisions and practices impact population health, and public health policies and practices impact how businesses operate. Mutually beneficial partnerships between these entities can help meet a business' core needs by leveraging a company's resources and offering actionable information to improve operations and increase impact. For academics, partnering with industry offers an opportunity to translate rigorous public health research into action, and to reach larger, more diverse audiences. To engage in meaningful academic-industry partnerships that both meet industry needs and academic goals, Boston University School of Public Health (BUSPH) established idea hub, a team of dedicated relationship managers that facilitate partnerships that align with faculty research. This piece describes the systems and processes idea hub developed to ensure these partnerships align with public health values, and provides an example of a successful collaboration with Ernst & Young, LLP (EY). With the right mission alignment, transparency, and open communication, academic-industry partnerships enhance the scholarly pursuits of faculty and advance public health interventions and initiatives through industry partners.

## Establishing transparent, mission-aligned partnerships

While academic-industry partnerships are well-established in medicine and engineering, they are relatively rare in public health. This stems from historic mistrust between these two entities: business views public health as overly regulatory, while public health is wary of entities like the alcohol and opioid-producing industries that harm the health of populations. But effective partnerships can benefit both parties (1, 2).

For industry, integrating public health thinking and research has a variety of potential benefits:

- *Meeting core business needs*. The COVID-19 pandemic demonstrated that public health impacts all of us, including business. Integrating public health thinking into strategic plans, products, and operations makes good business sense. Public health thinking can take many forms, including supporting employee health and wellbeing to boost retention and satisfaction, or evaluating a program or service to ensure it is effective and cost-efficient.
- *Impactful, credible research*. Public health research is designed to be practical and actionable. Companies can use the results of these studies to make informed decisions while also associating their brand with a reputable academic institution.
- *Expertise and diversity of thought*. Academics are required to stay current on new research and methods within their fields, bringing discipline-level expertise to research projects and diversity of thought and training to these partnerships.
- *Improving the employee pipeline*. Schools of public health attract diverse, ambitious students who are often involved in research teams during their educational programs. These projects provide a natural pipeline for partner companies to attract a diverse workforce with public health training, including a critical understanding of the ways that systems at all levels of the socioecology perpetuate and reinforce health inequities.

For academics, there are many potential benefits of academic-industry partnerships in public health:

- *Impact*. In academia, we conduct research to have an impact, and to improve the health of populations. Industry partnerships move us beyond the ivory tower of academia to entities that heavily influence how populations live and work.
- *Growth*. Industry partnerships spread public health thinking to the employees at collaborating organizations. Employee engagement has a ripple effect, leading to additional academic-industry partnerships and larger networks for attracting students to public health educational programs.
- *Scale*. Conventional academic research, through traditional funding mechanisms, proposes small, incremental changes and is published and presented largely for academic audiences. Partnerships with industry have the potential for larger impact: sharing rigorous public health research with large, non-public health audiences, integrating public health frameworks and values into how products and services are designed and delivered.
- *Novel areas of research*. Industry partnerships open different funding avenues, allowing public health academics to move beyond the traditional disease-focused perspective of funders like the National Institutes of Health (NIH) to novel and relevant areas of public health. For example, the project highlighted below focuses on how health inequities are and are not represented in women's health websites, an important area of research given that most women use the internet to look up health information, but one that would not typically be funded by NIH or other large funders.
- *Speed*. The typical start-up time for industry-funded partnerships is weeks, compared to 12- to 18-months with traditional funding.
- *Diversity*. Partnering with industry promotes diversity of thought, which spurs innovation and creative solutions thinking. Industry partnerships bring together different mindsets and interdisciplinary training to solve a public health problem.
- *Training the next generation*. These partnerships provide excellent real-world experiences for our students and trainees.

As mentioned earlier, some public health faculty are hesitant to engage with industry partners due to past harms by alcohol, tobacco, and opioid manufacturers, among others. Given this context, academic-industry partnerships at BUSPH go through an extensive vetting process to ensure both the partner organization and the project align with the school's mission and values. We ensure there is operational alignment, particularly around the company's expectations of timeline compared to a traditional academic timeline. idea hub works closely with faculty to ensure the project aligns with their research interests and that their past and future academic pursuits will be protected through formal contracting.

Idea hub at Boston University School of Public Health (BUSPH) facilitates partnerships with for-profit corporations that advance the science of public health while also expanding the impact of population health research. Identifying, vetting, and fostering relationships with industry takes time and effort; idea hub focuses on those relationships so faculty can focus on conducting research. idea hub also funds innovation grants and connects faculty with tech transfer and licensing services.

## The BUSPH-EY collaboration: putting the principles into practice

With an understanding that many women seek health information online, Ernst & Young, LLC partnered with BUSPH faculty to conduct a formative study on how women's health information is provided online (the Online Environments and Women's Health project). This project used an iterative in-depth review and coding process to assess whether a sample of women's health websites addressed the needs of marginalized women and determine what opportunities exist in online environments to mitigate health inequities for women across the life course.

Before beginning the project, we evaluated the mission alignment between the two entities. EY's public health group and BUSPH share a mission to advance health equity and improve the health of populations worldwide. EY argues that advancing health equity—increasing opportunities for everyone to live the healthiest life possible, regardless of identity, experience, health, geography, or financial status (3)—makes sense for businesses, while BUSPH focuses on training public health professionals and generating and disseminating new science. Specific to this project, EY was interested in making the business case for supporting women's health. Women are the greatest consumers of online health information (4) and make the majority of healthcare decisions for themselves and their families (5). Having relevant, inclusive online information for consumers will benefit a business' bottom line. The faculty PIs were aware of these different underlying motivations and received a detailed briefing on EY so they could make an informed decision about engaging in the project.

The first phase of our work was establishing a common understanding, as we brought together a research team with varied backgrounds and perspectives. The BU collaborators do not have experience working in industry, nor do the EY partners have experience working in academia. Two team members, one from each organization, have prior experience with academic-industry collaboration. The lead EY collaborator holds a doctorate in public health. We began with establishing an understanding of health equity (6) and the literature on digital health equity (7, 8). We had a shared understanding of the diversity of the populations of interest. “Women” and “women's health” are not one size fits all. Women's experiences and health information needs vary by socioeconomic position, geography, education, LGBTQ+ identity, disability status, and overall health literacy. As such, women's health-oriented websites are challenged to be accessible and relevant across a wide range of characteristics, to ensure end-users see themselves reflected and able to engage with the site's content. The persistent health inequities experienced by marginalized women highlight the need to consider these women specifically when designing online content to be relevant and valuable.

The BUSPH faculty designed an inclusion/exclusion framework for the study. Since this was a brief, formative study, the search was limited to US-based websites written in English and focused on the health of adolescent girls or women (cisgender and transgender women). By design, the definition of health was broad, but excluded topics that were not explicitly health (e.g., parenting or healthy eating/recipes alone), and the search framework excluded oral health, cosmetics, and elective procedures. We searched for key words related to health equity, including equity/inequity, disparity, diversity, inclusion, and marginalized (3). The first wave of data collection was then conducted by an EY research team, and vetted by the BU faculty. We assessed use of inclusive language, like use of they/them pronouns and narratives offered from different perspectives. We assessed selected indicators of website accessibility, including translation options and alt text for images.

The project brought something new to the field: a practical approach to integrating health equity frameworks into website design, using both a business and public health perspective. We found that the 75 websites evaluated did not prioritize health equity-oriented language, content, or images, and proposed actionable steps for how organizational leadership can move in this direction.

One of the most interesting lessons from the project was how the varied perspectives of the interdisciplinary team made the collaboration stronger, and validated the work such that the results resonated from both a public health and business perspective. This collaboration was successful because our team communicated regularly, transparently, and respectfully. We discussed each logistical detail, from how often to meet and how to organize our meetings to authorship order and project responsibilities. These early, open lines of communications helped overcome one of the greatest challenges in industry-academic projects: different organizational norms around credit and timeline.

In many ways, this was an unusual project. Industry partners do not typically hold doctorates in public health and the research is not typically conducted as a joint venture. At the outset of the project, the team agreed to share data resources after the project was completed, publish jointly, and each team could publish separately should they wish following the project. Understanding the potential for real or perceived conflict of interest, the BUSPH faculty determined a white paper would be the preferred final publication. In most idea hub collaborations, the faculty conduct research independently from the industry partner and faculty publish independent results. In this case, the project was set up to be a full collaboration at all stages from idea generation to design to dissemination. This was possible given the team's shared understanding from the start that the goal of this descriptive study was to be informative to EY business partners and that the strength of the collaboration was in combining the skills and knowledge of the EY team (e.g., writing for a business audience, understanding of how public health goals and business goals align) and BUSPH team (e.g., public health priorities, theory, and methodology).

The final white paper, which is available on the BUSPH idea hub website (https://www.ideahub.org/successes/womenshealthonline/), is intended for business leaders and strategists who would value the shared contributions from industry and academia. EY shared the results with their global client base, a large, diverse audience outside of academic public health. To date, dissemination has been through social media and the research team is brainstorming additional avenues for dissemination. For the BUSPH faculty, the project developed a new research framework for analyzing websites related to women's health. This framework and data produced through this collaboration will be available to the faculty for future research, both in collaboration with EY and independently.

## Discussion

Academic-industry partnerships in public health provide many opportunities to enhance our understanding of health inequities and how to improve population health. The Online Environments and Women's Health project was mutually beneficial: EY used this information to educate their global client base on how to improve women's health information by using a health equity lens in website design and communications; BUSPH faculty were afforded the opportunity to conduct novel scholarship in a relevant topic area not typically supported by traditional public health funding streams. The project achieved its goal to build awareness around the varied experiences and health needs of women, and why focusing on marginalized communities should be a priority for business. With the right mission alignment, transparency, and open communication, these partnerships provide the opportunity to enhance the scholarly pursuits of faculty and advance public health through industry partners.

## Author contributions

VE produced the first draft. SG, MF, AG, VE, and KN contributed to the design and execution of the cornerstone example mentioned in this piece, the online environments, and women's health study. All authors contributed to the manuscript revision, and read and approved the submitted version.
